# Screening of Candidate Bioactive Secondary Plant Metabolite Ion-Features from *Moringa oleifera* Accessions Associated with High and Low Enteric Methane Inhibition from Ruminants

**DOI:** 10.3390/metabo12060501

**Published:** 2022-05-31

**Authors:** Addisu Endalew Zeru, Abubeker Hassen, Zeno Apostolides, Julius Tjelele

**Affiliations:** 1 Department of Animal Science, University of Pretoria, Pretoria 0002, South Africa; nardosadd@gmail.com; 2 Department of Biochemistry, Genetics and Microbiology, University of Pretoria, Pretoria 0002, South Africa; zeno.apostolides@up.ac.za; 3 Range and Forage Sciences, Agricultural Research Council (ARC), Pretoria 0002, South Africa; jtjelele@arc.agric.za

**Keywords:** accession, methane inhibition, secondary plant metabolites, *m*/*z* ion-features, relationship, ruminants

## Abstract

This study evaluated the relationship of secondary bioactive plant metabolite ion-features (MIFs) of *Moringa oleifera* accessions with antimethanogenesis to identify potential MIFs that were responsible for high and low methane inhibition from ruminants. Plant extracts from 12 Moringa accessions were evaluated at a 50 mg/kg DM feed for gas production and methane inhibition. Subsequently, the accessions were classified into low and high enteric methane inhibition groups. Four of twelve accessions (two the lowest and two the highest methane inhibitors), were used to characterize them in terms of MIFs. A total of 24 samples (12 from lower and 12 from higher methane inhibitors) were selected according to their methane inhibition potential, which ranged from 18% to 29%. Ultra-performance liquid chromatography-mass spectrometry (UPLC-MS) and untargeted metabolomics with univariate and multivariate statistical analysis with MetaboAnalyst were used in the study. Although 86 MIFs showed (*p* < 0.05) variation between higher and lower methane inhibition groups and lay within the detection ranges of the UPLC-MS column, only 14 were significant with the volcano plot. However, Bonferroni correction reduced the candidate MIFs to 10, and their R^2^-value with methane production ranged from 0.39 to 0.64. Eventually, MIFs 4.44_609.1462 and MIF 4.53_433.1112 were identified as bioactive MIFs associated with higher methane inhibition, whereas MIF 9.06_443.2317 and 15.00_487.2319 were associated with lower methane inhibition with no significant effect on in vitro organic matter digestibility of the feed. These MIFs could be used by plant breeders as potential markers to develop new *M. oleifera* varieties with high methane inhibition characteristics. However, further investigation on identifying the name, structure, and detailed biological activities of these bioactive metabolites needs to be carried out for future standardization, commercialization, and application as dietary methane mitigation additives.

## 1. Introduction

Methane (CH_4_) is the second most important greenhouse gas next to CO_2_, and 35–40% of its anthropogenic emission is contributed by ruminant animals that rely on low-quality roughage feed [1,2]. The animal production sector has been highly threatened due to its global warming potential and negative effect on animal productivity. Hence, several studies were conducted [3,4,5,6,7,8] and the development of suitable mitigation strategies have been compelled to minimize the shadowing effect to the sector. However, the established long-term reduction effect has not been produced as expected. Recently, most enteric methane abatement strategies have focused on feeding and feed additives. The use of antibiotics and synthetic chemical feed additives is becoming less popular globally and in EU countries in particular, because of the concerns with long-term residual effects on human health [9]. Hence, the global scenario has shifted from the use of synthetic antibiotics to natural plants and their extracts to produce organic animal products free from dietary antibiotic residues [10,11,12]. Medicinal plants such as *M. oleifera*, which are rich in bioactive metabolites, are relatively cheaper and safe to replace synthetic chemical feed additives [3,7,13,14,15,16,17]. The potential of medicinal plants to reduce CH_4_ has been associated with the presence of non-nutritive secondary plant metabolites (SPMs), which have developed over years as a survival and defense mechanism against herbivores, pests, microorganisms, invaders, grazers, and parasites [18,19]. Studies would have generally associated the microcidal or microstatic action of SPMs with the capacity to form irreversible complexes with cholesterol in the microbial cell membrane [20], and interfere with the bacterial cell membrane to disintegrate membrane structures that cause ion leakage and cell lysis, which ultimately reduced methane emission [3]. Thus, exploring different aspects of the biologically active metabolites in medicinal plants such as *M. oleifera* is very crucial and has been a highly demanded research field [21].

Several studies have tried to profile the phytochemical constituents of *M. oleifera* due to its multipurpose functions [22,23,24,25,26,27]. However, the composition, concentration, and the bioactivities of SPMs may differ among plant species, varieties, ecotypes, cultivars, and individual plants of the same species and even among plant parts [28]. Studies have shown that leaf extracts of three varieties of *Labisa pumila* Benth illustrated different total phenolic and flavonoid content, and antioxidant activities [29]. Likewise, the genotype is stated to be the main factor for the differences in the bioactive compounds and bioactivity in green coffee and berries [30,31]. *Moringa oleifera* accessions grown in Chad, Algeria, and Haiti showed substantial variation in total polyphenols and salicylic and ferulic acids [27]. Similarly, Moringa accessions grown in China and India were different in composition and concentration of plant components [32]. The *M. oleifera* leaves collected from Gauteng, Limpopo, and Mpumalanga provinces in South Africa also exhibited different nutrient contents, metabolite profiling and antioxidant activities [33]. Hence, these variations might be attributed to the genotype and growing environment or the effects of their interactions. However, many of these studies did not show variations in terms of metabolites between accessions when they were grown in the same environment and were used as a source of plant extracts for rumen modulation and antimethanogenesis.

The inclusion of flavonoids such as flavone, myricetin, naringin, rutin, quercetin, and kaempferol at 4.5% *w*/*w* dry matter (DM) of the substrate decreased in vitro CH_4_ production by 5 to 9 mL/g DM [34]. Luteolin-7-glucoside showed more promising results on CH_4_ and NH_3_ inhibition without compromising the fermentation efficiency relative to quercetin, epicatechin, isoquercetin, catechin, epigallocatechin, epigallocatechin gallate, and gallocatechin [35]. However, the tannic acid, gallocatechin, and epigallocatechin gallate decreased the gas production and *IVOMD* compared with the negative control [35]. Depending on the structure and functional group of the SPMs, their effect on antibacterial, antifungal, antiviral, antiprotozoal, and CH_4_ production might be inhibition or stimulation [36,37,38,39]. Strongly inhibited growth and metabolism of rumen microbes were obtained from the oxygenated monoterpene SPMs, whereas slight inhibition and occasional stimulation of rumen microbial activities were reported in the hydrocarbon monoterpene SPMs [40]. Plant metabolites with functional groups of phenolic acids, phenols, and terpenoids also showed strong antimicrobial activities [22,41,42,43], which might influence the antimethanogenic potential of the extracts in diverse ways. Studies on the relationship of plant metabolites with antimethanogenic potential using Moringa as a model medicinal plant helped to establish applicable knowledge about the active metabolites responsible for antimethanogenesis. These active metabolites can also be used as a marker for the selection of commercial varieties that will be used as a source of plant extracts to mitigate enteric methane from ruminants. However, many studies did not describe this aspect of medicinal plants and their components at the metabolite level, except for some that showed the use of crude plant extracts [16,44,45,46]. Therefore, this study intended to establish the relationship between secondary plant metabolites’ *m*/*z* ion-features with the in vitro methane inhibition characteristics of Moringa accessions. The intention was to identify potential *m*/*z* ion-features that could be used as markers to select breeding varieties with high methane inhibition characteristics. These could then be used as dietary additives to the ruminants’ feeding system.

## 2. Materials and Methods 

### 2.1. Plant Materials and Preparation of Crude Extracts

Eleven *M. oleifera* and one *Moringa stenopetala* accession were grown from October 2018 to February 2019 in the subtropical climate of Pretoria at Roodeplaat experimental site of the Agricultural Research Council (ARC), Pretoria in South Africa, which is located at 25°44′30′′ S, 28°15′30′′ E. Prior to this study, plant extracts from these accessions were evaluated for in vitro gas production and methane-reducing potential when used as dietary additives. The accessions were classified as low and high methane inhibition groups based on their CH_4_ inhibition potential. Subsequently, four accessions—that is, two accessions that contained 12 samples that exhibited the lowest antimethanogenic effect, and another two that constituted 12 samples of the highest antimethanogenic potential—were selected for this metabolomics study [47] (unpublished). Two samples per plot or six samples per accession were collected at five months after the Moringa accessions had been transplanted to the field. This coincided with the end of the summer season for 2019. 

The leaves were freeze-dried for about five days and milled to a 1 mm sieve size in a milling machine. About 50 g of the dried leaf powder was suspended in 500 mL of methanol at a ratio of 1:10 (mass/volume) [48] for 96 h in a shaker. The mixture was filtrated and extracted through a 150 µm aperture sieve (Vickers sieve, Durban, South Africa). The solution containing the extract was placed in fume chambers until it was semidried for about 48 h. The semidried extracts were then dried until constant weight was attained and kept at 4 °C for further use.

### 2.2. Determination of In Vitro Total Gas, Methane, and Organic Matter Digestibility

The total gas, antimethanogenic potential, in vitro organic matter digestibility (*IVOMD*) of *Eragrostis curvula* hay, and CH_4_/*IVOMD* treated with these Moringa accession leaf extracts were determined in a previous study and imported into this part of the study [47] (unpublished). Buffer, macro-mineral, and micro-mineral solutions were prepared according to simplified in vitro medium preparation procedures before the incubation day and kept in the fridge at 4 °C [49,50]. Hence, the buffer solution constituted ammonium bicarbonate (NH_4_HCO_3_) and sodium bicarbonate (NaHCO_3_), while the macromineral solution was prepared from sodium hydrogen phosphate dibasic (Na_2_HPO_4_), potassium phosphate monobasic (KH_2_PO_4_), and magnesium chloride hexahydrate (MgCl_2_ × 6H_2_O). The micromineral solution was composed of calcium chloride dihydrate (CaCl_2_ × 2H_2_O), manganese chloride tetrahydrate (MnCl_2_ × 4H_2_O), cobalt chloride hexahydrate (CoCl_2_ × 6H_2_O), and ferric chloride hexahydrate (FeCl_3_ × 6H_2_O) [49]. Early in the morning of incubation day, the prepared micro-mineral, macro-mineral, buffer, and resazurin (0.1% *w*/*v*) solutions were mixed with tryptone and distilled water. Then, the mixed solution was bathed at 39 °C and continuously bubbled with CO_2_ until the incubation process was completed to keep the anaerobic condition. Before morning feeding of the incubation day, the rumen fluid was collected from three ruminally cannulated Pinzyl (Pinzgauer cross Nguni) steers and adapted for 14 days, following procedures approved by the Animal Ethics Committee of the University of Pretoria (No. EC039-18), South Africa. L-cysteine and sodium sulphide nonahydrate (Na_2_S × 9H_2_O) were added to the medium 10 min before the addition of rumen fluid [50]. When the solution was sufficiently reduced, the rumen fluid was filtered through four layers of cheesecloth and mixed with a buffer medium at a 15:25 mL rumen fluid-to-medium ratio. The dried extract was re-dissolved with distilled water and applied with a 50 mg/kg DM feed as recommended in a previous study [16] except for the blanks and the control. Then, 40 mL of the inoculum was added to all the bottles and incubated with an incubator or Inco-shake set at 39 °C and 120 revolutions per minute (rpm).

The total gas produced during incubation was measured with a pressure transducer attached to a digital data logger at 3, 6, 12, 24, and 48 h of incubation, and recorded in pounds-force per square inch (psi) [51]. The gas pressure was converted to ml as V_x_ = V_j_P_psi_ × 0.068004084, where V_x_ is the gas produced at 39 °C in mL; V_j_ is the headspace of the incubation bottle in mL; and P_psi_ is the pressure recorded by the gas monitor system software [52].

Similarly, CH_4_ samples were taken at 3, 6, 12, 24, and 48 h of incubation and analyzed by gas chromatography (GC) (8610C, SRI Instruments GmbH, Bad Honnef, Germany) equipped with a flame ionization detector. The CH_4_ area obtained from the GC was converted into parts per million (ppm) using a standard curve and then changed into a percentage. Next, the CH_4_ concentration was converted to ml by multiplying the total gas produced (mL) with the percentage of CH_4_ in the sample as CH_4_ (mL) = total gas produced (mL) × % CH_4_ concentration. Eventually, the antimethanogenic potential of the plant extract was expressed as a mL/g DM incubated feed [46]. In addition, methane inhibition potential was converted to a percentage compared with the control, and the accessions were classified into lower and higher methane inhibition groups. Of the 12 accessions, 4 accessions (A3 and A11 to higher, and A1 and A2 to lower methane inhibition) containing 24 samples were selected (Table 1) to characterize the *m*/*z* ion-features of Moringa accessions with methane inhibition and identify the MIFs responsible for their antimethanogenic potential.

In addition, the *IVOMD* was determined with the modified two digestion phase techniques [53,54] and imported to this part of the study to understand the relationship of these MIFs with feed digestibility. In the first phase of the incubation, 200 mg substrate feed, *E. curvula* grass hay, extracts of the accessions (50 mg extract/kg DM feed), artificial saliva solution, and urea and rumen fluid were incubated under anaerobic conditions for 48 h at 39 °C using a test tube, which was fitted with modified stoppers. Artificial saliva was prepared from potassium chloride (KCl), disodium hydrogen phosphate (Na_2_HPO_4_), sodium bicarbonate (NaHCO_3_), sodium chloride (NaCl), magnesium sulphate heptahydrate (MgSO_4_·7H_2_O), and calcium chloride (CaCl_2_) before the incubation day and kept in the refrigerator until use. In the second phase, an acid and pepsin-containing solution were prepared from 20 mL of 32% HCl and 8 g of pepsin by dissolving them in 2000 mL of distilled water. Then, 20 mL of this solution was added after gently decanting the liquid on the top of the tubes and incubating for another 48 h at 39 °C. After 96 h of total anaerobic incubation, the residual plant materials were oven-dried at 100 °C for 18–24 h, ashed, and weighed, and the percentage *IVOMD* was calculated as follows:

$$\%\;IVOMD=\frac{OM\;of\;feed\;sample-\left(undigested\;residue-blank\right)}{OM\;of\;feed\;sample}$$

### 2.3. UPLC-MS Data Analysis

To study metabolomics, about 100 mg of the crude extract used to determine in vitro methane was re-dissolved with 10 mL of 70% methanol (10 mg/mL concentration) in the Department of Chemistry, University of Pretoria, South Africa. The mixture was then vortexed and centrifuged for 10 min. Then, 10 µL of the sample supernatant was taken and diluted again with 990 µL of 70% methanol. About 1 mL of solution (0.1 mg/mL) was filtered with 0.2-micron syringe filters and transferred to the injection vial of UPLC-MS for analysis. The UPLC-MS analyses were carried out with a Waters Synapt G2 quadrupole time-of-flight (QTOF) MS connected to a Waters Acquity UPLC (Waters, Milford, MA, USA).

The samples with the injection vials were subjected to both the positive and negative ion mode detector of the UPLC-MS. The untargeted metabolomics data were transferred to Microsoft Excel and a MetaboAnalyst 5.0 system for univariate and semi-quantitative multivariate statistical analysis. After clustering the accessions with their antimethanogenic potential as high or low CH_4_ inhibition, two accessions (A3 and A11), which contained 12 samples and showed the best in vitro CH_4_ inhibition (hereafter ‘high’), and another two accessions (A1 and A2), which comprised 12 samples and recorded the lowest CH_4_ inhibition, were selected as ‘low’ methane inhibitors. Then, before the metabolomics analysis, the low and high CH_4_ inhibition labelling of the samples was added to the UPLC-MS data. The MIFs in the column regeneration step (after 17 min) and the void volume (0–2 min) of the detector were poorly resolved from each other and ignored in all statistical analyses. Thus, the focus of the study was MIFs detected in the chromatogram between 2 and 17 min. A data pre-processing strategy for metabolomics was carried out to reduce the masking effect in data analysis. The values that were missing because of the limits of quantification in the detector were managed with a modified 80% rule. Thus, variables were excluded when the proportion of non-missing elements accounted for less than 80% in each biological group [55]. Besides, as recommended in MetaboAnalyst 5.0, the quality of LC/MS data was controlled by 20% relative standard deviation (RSD) and the ion-features that showed low repeatability or greater than 20% RSD were removed before analysis. Next, the UPLC-MS data were subjected to continuous univariate and multivariate statistical analysis, such as a *t*-test, fold change, volcano plot, and principal component analysis (PCA) with MetaboAnalyst 5.0. Initially, PCA was performed to explore the maximum variation to obtain the overview and classification. The volcano plot visualized the predictive component loading and identified important MIFs by the fold change and *t*-tests. After analyzing the significant variation of each MIF intensity by the *t*-test (*p* < 0.05), the Bonferroni correction was applied to minimize the multiple testing problem or false discovery rate [56]. The relationship (R^2^-value) of MIF intensity with methane production was determined with scatter plot analysis. 

### 2.4. Statistical Analysis 

One-way analysis of variance (ANOVA) and least significant difference (LSD) for randomized complete block design (RCBD) were conducted to determine the variations in terms of total gas, CH_4_ production, and *IVOMD* among these accessions using SAS version 9.4 [57]. To investigate the variations and relationships of MIFs with antimethanogenic potential, continuous semi-quantitative univariate and multivariate statistical analyses such as *t*-tests, fold change, the volcano plot, and PCA were carried out with MetaboAnalyst 5.0. The relationship (R^2^-value) between MIF intensity and CH_4_ production, total gas production, and *IVOMD* was determined with scatter plot analysis. The Bonferroni correction was also calculated and applied at the corrected *p* ≤ 6.25 × 10^−3^ in the negative ion mode and *p* ≤ 6.25 × 10^−4^ in the positive ion mode [56]. 

## 3. Results

### 3.1. In Vitro Methane Inhibition 

**Figure 1:**
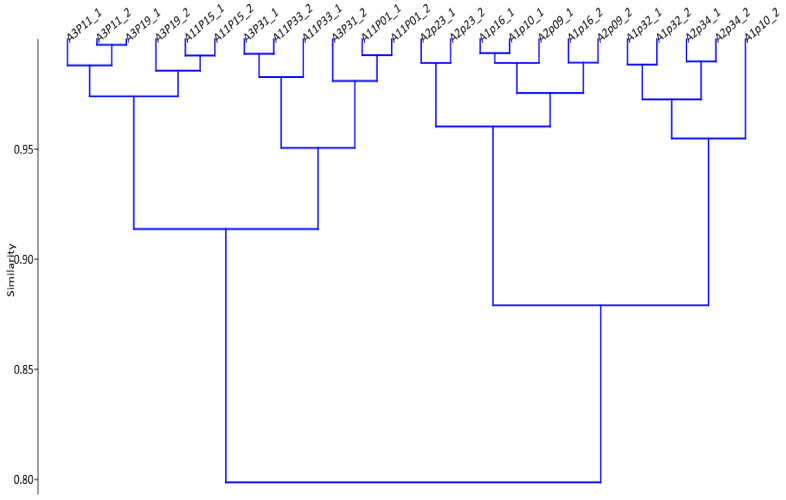
Hierarchical cluster analysis of Moringa accession samples using total methane produced (mL/g DM) from *Eragrostis curvula* hay treated with their extracts and the percentage methane inhibition potential. A: accession code of the samples; P: plot number of the samples collected in the field. A1P10_1 refers to accession 1, plot 10, sample 1; A1P10_2: accession 1, plot 10, sample 2, etc.

The substrate feed, *E. curvula* hay, which was used for in vitro fermentation and methane determination, was a relatively poor-quality roughage that contained about 93.08% dry matter (DM). Its composition was 8.57% crude protein (CP), 80.02% neutral detergent fibre (NDF), 41.01% acid detergent fiber (ADF), 8.34% acid detergent lignin (ADL), and 3.92% ash content on a DM basis. The total gas and total CH_4_ and CH_4_ yield per unit of organic matter (OM) digestibility of *E. curvula* hay treated with the leaf extracts were significantly *(p* < 0.01) varied in the study. Their total methane production and methane yield per unit of OM digestibility ranged from 4.52 mL/g DM (A11) to 5.23 mL/g DM (A1) and 7.61 (A3) to 8. 81 mL (A1) per OM digestibility, respectively. Thus, the total methane production and methane yield per unit of OM digestibility were significantly (*p* < 0.01) decreased in all accession extracts compared with the CH_4_ produced in the control (6.37 mL/g DM; 11.65 m/g OM). Compared with the control, the recorded antimethanogenic potential (% CH_4_ inhibition) of the accessions ranged from 18% (A1) to 29% (A11). Thus, the hierarchical cluster analysis (HCA) grouped the study samples into two broad cluster trees as high and low CH_4_ inhibition with 80% similarity (Figure 1) using their antimethanogenic variation. The higher and lower CH_4_ inhibition categories of the samples in their antimethanogenic potential (Figure 1; Table 1) involved drawing an arbitrary line through a continuous distribution. Thus, the 12 samples of *M. oleifera* A3 (07633) and A11 (Pretoria) lay in a higher CH_4_ inhibition cluster tree, whereas the other 12 samples of A1 (bulk) and A2 (07229) belonged to the lower CH_4_ inhibition cluster tree (Figure 1). In addition, the *E. curvula* hay treated with extracts of these accessions exhibited the *IVOMD* of about 58% to 60%. None of the four accessions recorded a negative effect on feed OM digestibility, and showed small improvement compared with the OM digestibility of the control (55%). Thus, the variations in these parameters might be attributed to the differences in the active compounds in the extracts, which were expressed as SPM *m*/*z* ion-features. 

### 3.2. Characterizing the m/z Ion-Features of Moringa Accessions with Methane Inhibition 

**Figure 2:**
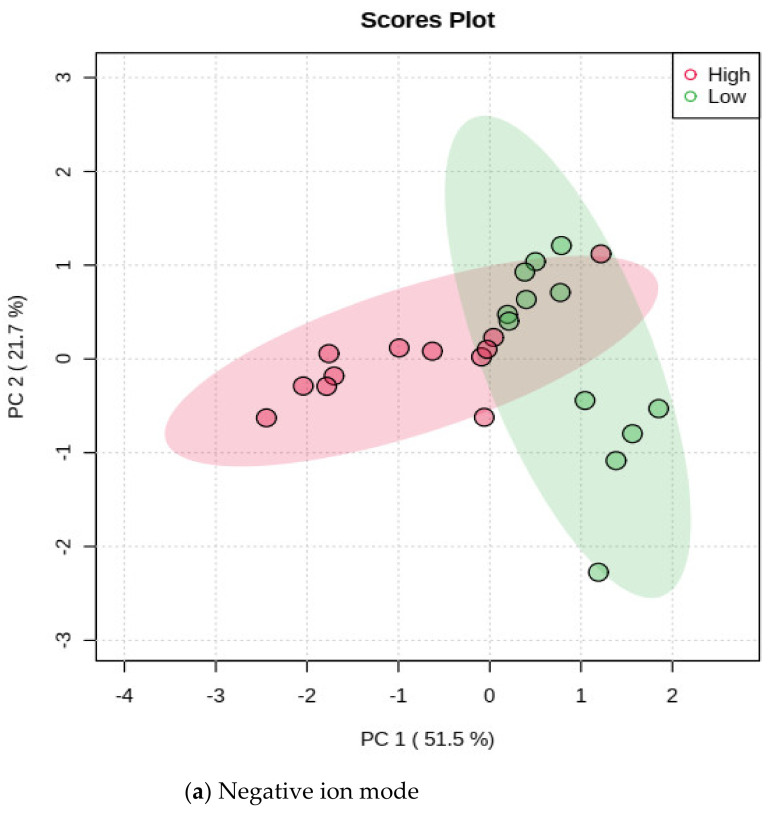

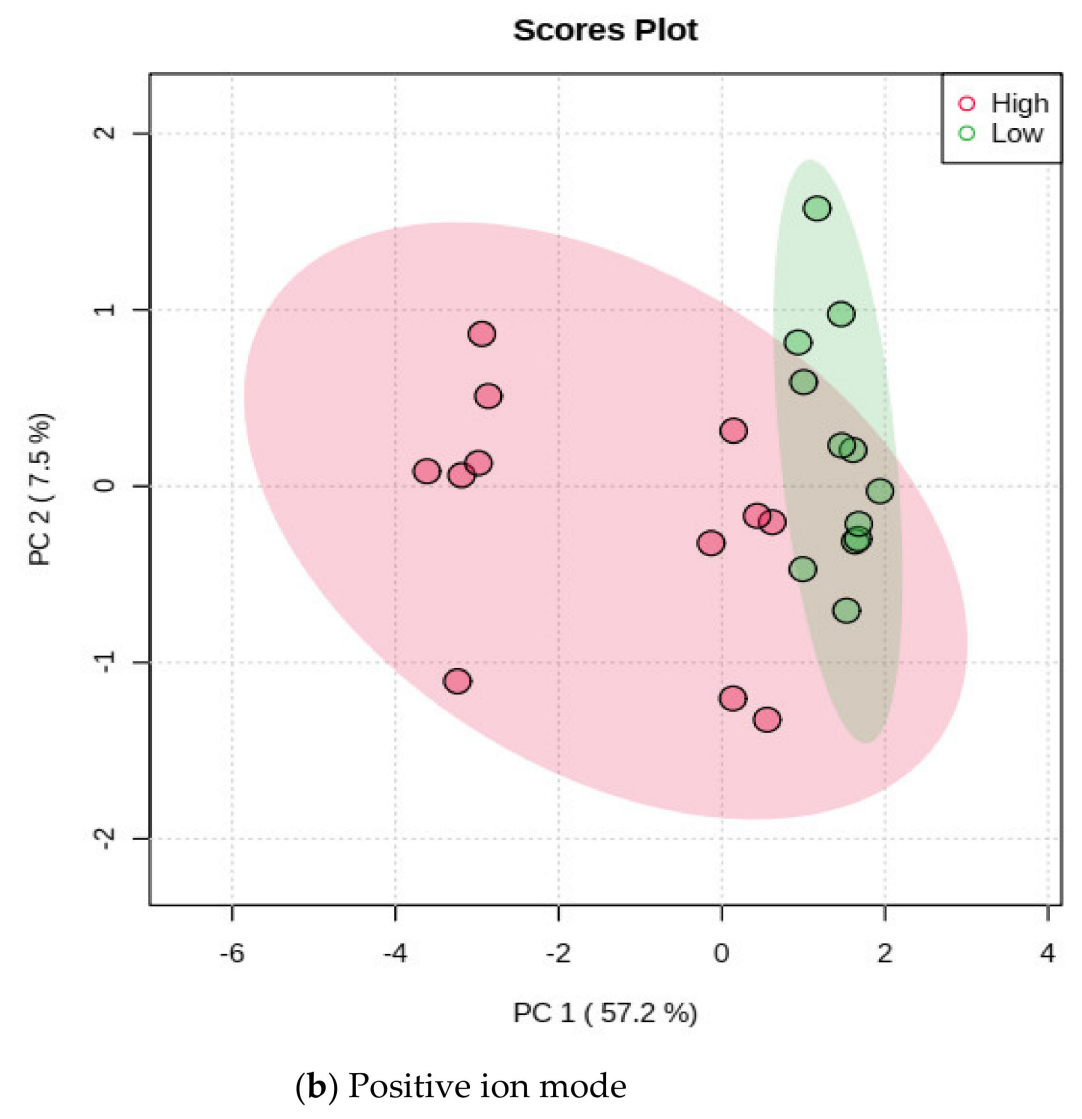
Principal component analysis score plot between the selected principal components that grouped the samples into high and low methane inhibition.

**Figure 3:**
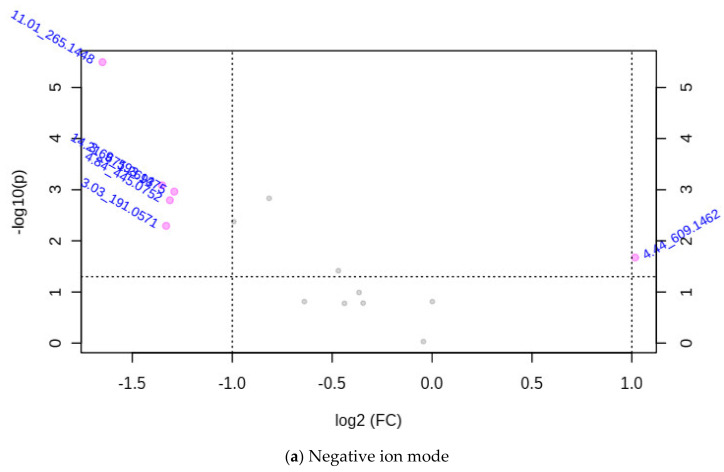

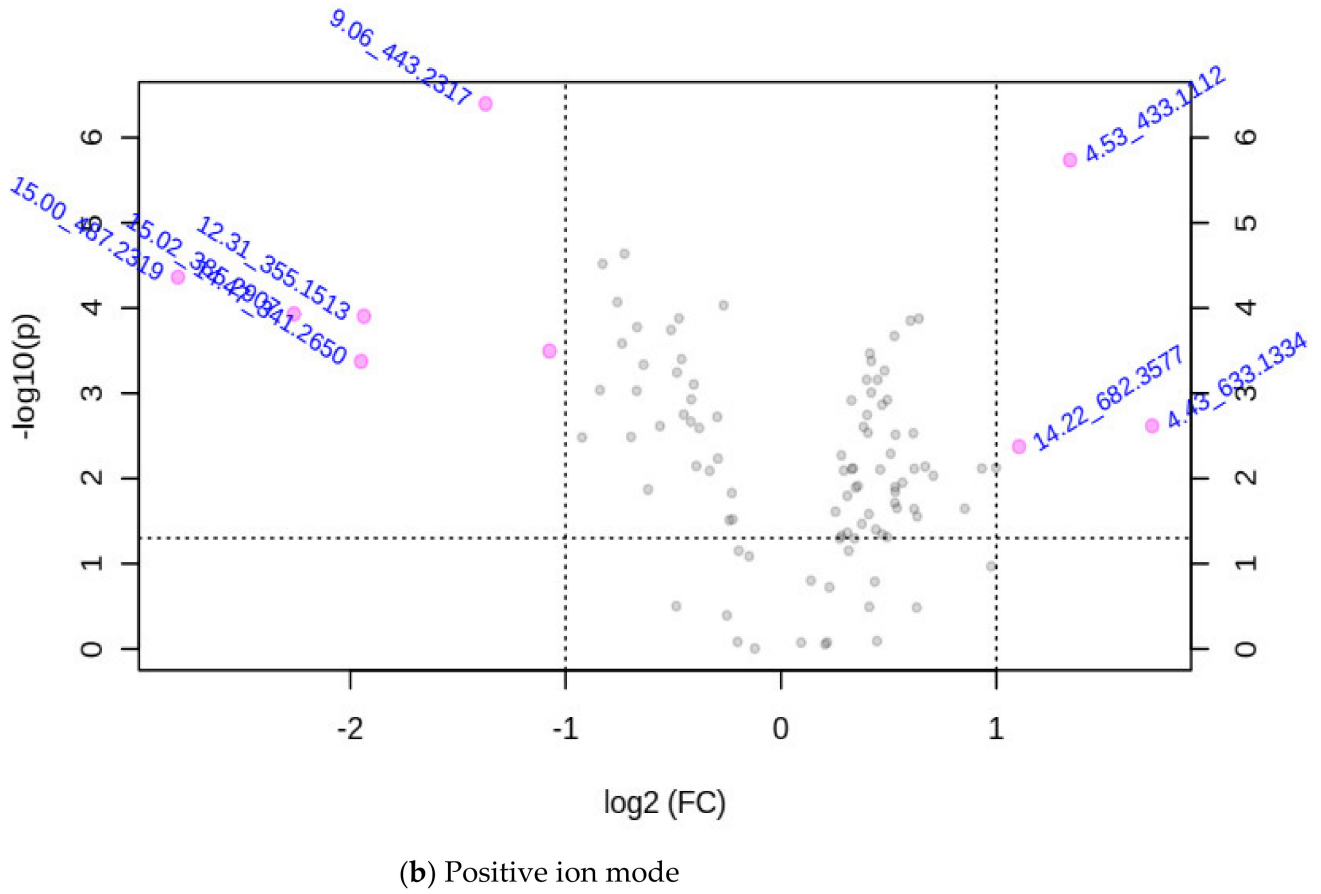
Important *m*/*z* ion-features selected by volcano plot with fold change threshold (x) 2 and *t*-tests threshold (y) 0.05.

**Figure 4:**
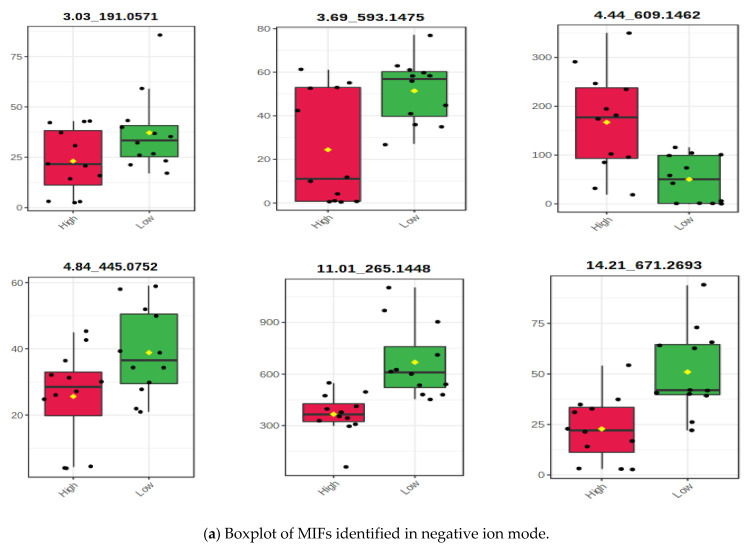

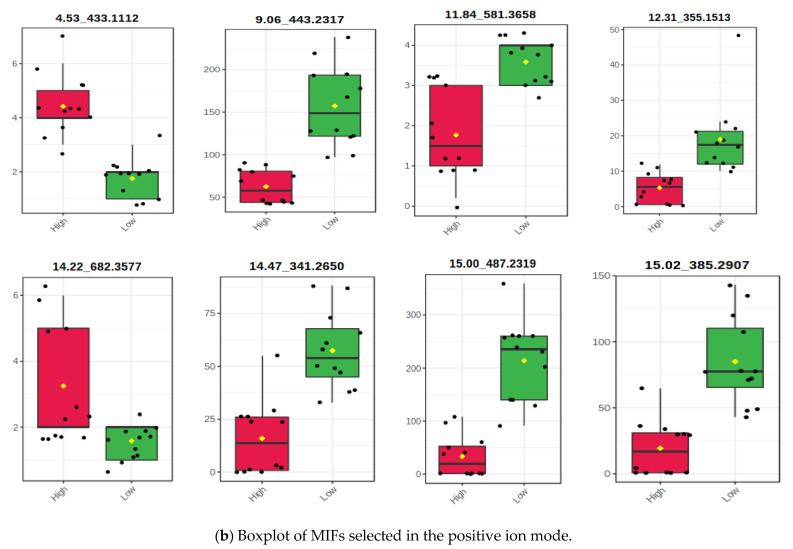
Boxplot of selected *m*/*z* ion-features. Black dots represent the abundance of the selected ion-feature from all samples. The red and the green boxes indicate the 95% confidence interval around the median of each group, defined as +/−1.58*/QR/sqrt(n), which can be used to evaluate differences between groups. If the boxes do not overlap, the medians are probably different. The mean concentration of each group is indicated with the yellow diamond. The direction of comparison is ‘high methane inhibition’/‘low methane inhibition’.

**Figure 5:**
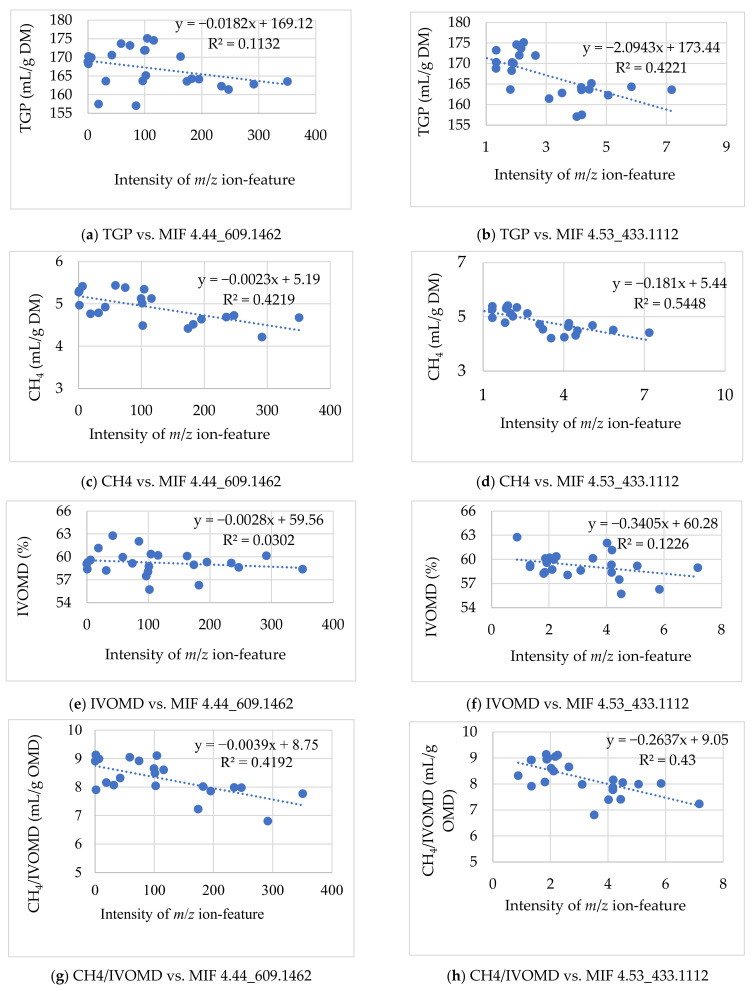
Scatter plot of selected *m*/*z* ion-feature 4.44_609.1462 and 4.53_433.1112 contributed to higher methane inhibition, which showed the relationship of *m*/*z* ion-feature intensities with in vitro total gas, methane production, organic matter digestibility, and methane yield per organic matter digestibility. TGP: total gas production (mL/g DM); *IVOMD*: in vitro organic matter digestibility; OMD: organic matter digestibility.

**Figure 6:**
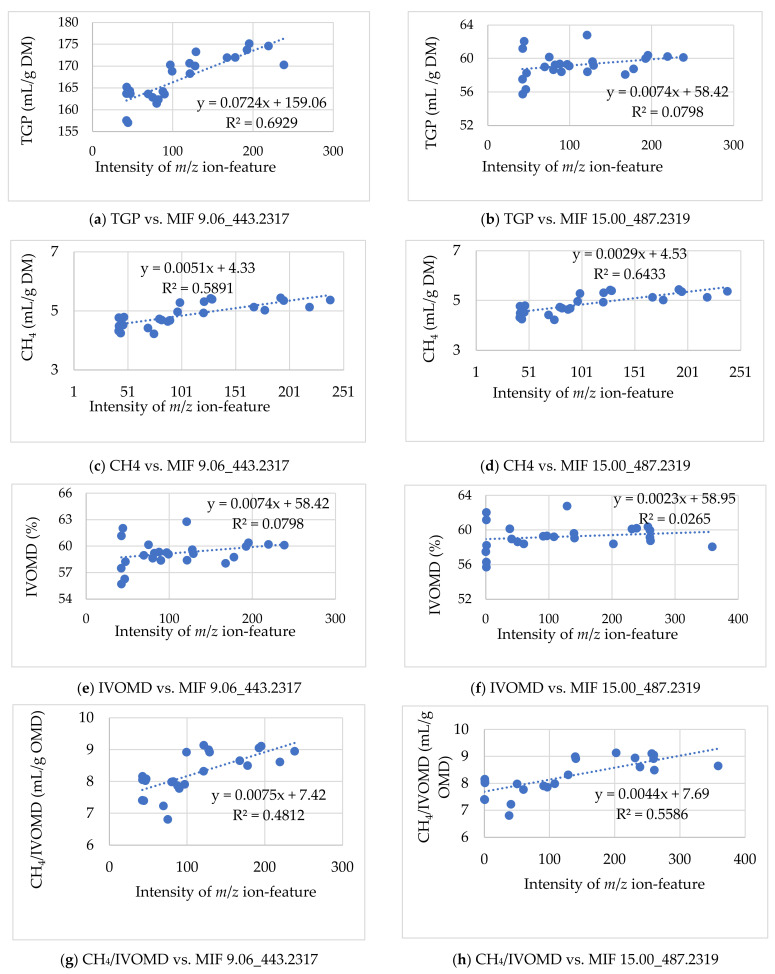
Scatter plot of the selected *m*/*z* ion-features 9.06_443.2317 and 15.00_487.2319 contributed to lower methane inhibition, which showed the relationship of their intensities with in vitro total gas, methane production, organic matter digestibility, and methane per organic matter digestibility across samples. TGP: total gas production (ml/g DM); *IVOMD*: in vitro organic matter digestibility; OMD: organic matter digestibility.

A significant decrease in methane production was obtained from all accessions of Morinaga leaf extracts compared with the control. Hence their antimethanogenic potential was significantly (*p* < 0.01) different. This antimethanogenic variation was expected because of the difference in composition and concentration of the plant components, which were represented as MIFs in this study. A large number of MIFs were detected at the initial step of the screening from the UPLC-MS data of the accessions (data not shown). However, only 161 (33 in the negative and 128 in the positive ion mode) were selected for MetaboAnalyst using the detection range of 2 to 7 min and a significant level of their intensity between the two methane inhibition groups before data processing (*p* < 0.05). The MetaboAnalyst output of the MIFs containing data obtained from UPLC-MS in the positive and negative ion mode is illustrated in Figure 2, Figure 3, Figure 4, Figure 5 and Figure 6. During the metabolite data analysis, the PCA (Figure 2) clustered the samples into high and low methane inhibition groups and explained about 64.7% in the negative ion mode and 73.2% in the positive ion mode of the total variations of the samples. The volcano plot (Figure 3) showed the importance of the MIFs in a model, which makes the difference between the two sample groups; whereas, the boxplot (Figure 4) illustrated the abundance of *m*/*z* ion-features in the samples, as well as a 95% confidence interval around the median of each group and their mean abundance in each group.

Among the 161 markers uploaded into MetaboAnalyst, eight MIFs in the negative ion mode and 78 MIFs in the positive ion mode were significantly different between high and low CH_4_ inhibition sample groups using the *t*-test at *p* < 0.05 (all data not shown). In addition, the volcano plot that is drawn from fold change threshold (x) 2 and the *t*-tests threshold (y) 0.05 selected 14 important MIFs (six in the negative ion mode and eight in the positive ion mode) that significantly varied between high and low inhibition samples (Figure 3). Using the Bonferroni correction, five MIFs in the negative mode (corrected *p* ≤ 6.25 × 10^−3^) and seven MIFs in the positive ion mode (corrected *p* ≤ 6.4 × 10^−4^) were significantly different between the two methane inhibition groups. Thus, the MIFs that showed significant variations in all the tests were promoted to the next stage of associating them with higher and lower methane inhibition potential. However, MIFs that did not vary significantly with Bonferroni correction, but showed a significant variation on the other tests, were considered in the selection because of their higher relative abundance for easy practical application and higher relationship value (R^2^-value) of the intensity with methane inhibition potential. 

The red circles/dots in this volcano plot represent ion-features above the threshold. The Bonferroni corrected value of the negative ion mode (0.00625) in the graph indicates 2.2, whereas the positive ion mode (0.00064) is 3.2. The direction of comparison is ‘high methane inhibition’/‘low methane inhibition’. The overlapped *m*/*z* ion-features in a and b are identical to the MIFs shown in box plots of Figure 4a,b below.

#### 3.2.1. Associating the m/z Ion-Features Contribution to High Methane Inhibition 

The potential candidate *m*/*z* ion-features responsible for high methane inhibition and their summary data are shown in Table 2. The relationship (R^2^-value) of MIFs intensity that allied to higher methane inhibition with CH_4_ volume, total gas, *IVOMD*, and methane yield per OM digestibility was calculated with a scatter plot (Figure 5). However, several MIFs seemed to associate with high methane inhibition characteristics of the samples, and most of them showed a lower relationship value and were not statistically significant in the subsequent analysis. Thus, after long screening steps, one MIF in the negative ion mode (*p* < 0.05) and two MIFS in the positive ion mode (corrected *p* ≤ 6.4 × 10^−4^) were selected as candidates for higher methane inhibition potentials of the accessions. The relationship value determined with scatter plot analysis between the MIF intensities with CH_4_ produced per DM feed incubated and CH_4_ yield per OM digested were 0.42 and 0.41 in MIF 4.44_609.1462, and 0.54 and 0.43 in MIF 4.53_433.1112, respectively (Figure 5). Besides, the Pearson correlation analysis correlated negatively and significantly (*p* < 0.01) for the MIFs 4.44_609.1462 and MIF 4.53_433.1112 intensities with total methane production and methane yield per OM digestibility (Table 2). In terms of the magnitude of ion-feature intensity, the MIF 4.44_609.1462 from the negative ion mode showed relatively higher abundance in higher CH_4_ inhibition sample groups compared with MIF 4.53_433.1112 and MIF14.22_682.3577 in the positive ion mode. However, MIF 4.53_433.1112 exhibited the highest R^2^-value (0.54) and correlation coefficient (−0.66). Hence, the higher signal strength obtained in MIF 4.44_609.1462 might provide an advantage for analysis and practical application, though concentration is not the sole determinant factor of their antimethanogenic potential. It was proven from MIF 4.53_433.1112, however, its level of intensity was low, and it exhibited a greater relationship (R^2^-value) with CH_4_ production than was observed in MIFs with greater intensity. This result indicated not only that metabolite concentration played a crucial role in their antimethanogenic potential, but that the specific action of the metabolites and their thresholds of maximum and minimum dose-dependent influence might also determine their biological activities, including antimethanogenesis. The potential MIFs identified for their contribution to higher CH_4_ inhibition were found in high and low methane inhibition sample groups of the study. The variations in their effect on the two groups might be attributed to differences in their concentration. However, several MIFs seemed to contribute positively to the higher CH_4_ inhibition potential of the samples with different levels of contribution. The three MIFs illustrated in Table 2, individually or combined, provided the most responsibility for the higher CH_4_ inhibition potentials of Moringa accessions. However, MIF 4.53_433.1112 showed significant variations after the Bonferroni correction at corrected *p* ≤ 6.4 × 10^−4^ and seemed to have the greatest responsibility for the higher inhibition of methane recorded in the study. However, MIF 4.44_609.1462 could be a promising candidate in the future because of its larger intensity, which might be easier for analysis and practical applicability. It also had a relatively good R^2^-value and correlation coefficient.

This study also associated these MIFs with other major fermentation characteristics of ruminant animals such as total gas production (TGP) and *IVOMD* to see their overall effect on fermentation kinetics and animal performances. Thus, the MIFs selected for their contribution to higher CH_4_ inhibition correlated negatively and significantly with methane production, TGP and CH_4_/*IVOMD*; however, MIF 14.22_682.3577 was not correlated significantly with CH_4_/*IVOMD* at *p* < 0.01 (Table 2). However, MIF 4.44_609.1462 and 4.53_433.1112 were not significantly (*p* > 0.05) correlated with *IVOMD*, except MIF 14.22_682.3577, which correlated negatively and significantly (*p* < 0.05) with *IVOMD*. Thus, the MIF 14.22_682.3577 is not the interest of this study because of its negative effect on digestibility and non-significant reduction of CH_4_ yield per unit of OM digestibility, which might decrease the animal’s performance during its application. The relationship scatter plot analysis between MIF 4.44_609.1462 and MIF 4.53_433.1112 intensities versus TGP (mL/g DM), which showed R^2^-values of 0.1132 and 0.4221 (Figure 5). Thus, the contribution to higher CH_4_ inhibition potential obtained in these ion-features with no effect on the OM digestibility of the tested feed significantly proved that it did not influence fermentation kinetics negatively or the productivity of the ruminant animals. However, its detailed bioactivity needs to be confirmed by future studies.

#### 3.2.2. Associating the m/z Ion-Features with Their Contribution to Low Methane Inhibition

Among the MIFs that showed significant variation between the two sample groups, most allied with the lower methane inhibition group (all data not shown). After analyses of the *t*-test, fold change, PCA, and volcano plot, these candidate MIFs, for their contribution to lower methane inhibition, are presented in Table 3. Among the MIFs detected in the UPLC column that were significantly (*p* < 0.05) different between higher and lower CH_4_ inhibition in the *t*-test, most MIFs intensity and total CH_4_ production were associated positively with lower methane inhibition characteristics of the samples with R^2^-values ranging from 0.03 to 0.64. However, the volcano plot and Bonferroni correction were used to explore the MIFs most responsible for lower CH_4_ inhibition. Only seven MIFs (one in the negative ion mode and six in the positive ion mode) were varied between the two sample groups at the corrected *p* ≤ 6.25 × 10^−3^ in the negative ion mode and *p* ≤ 6.4 × 10^−4^ in the positive ion mode. Thus, the Bonferroni correction reduced the candidates to seven and selected the MIFs that showed an R^2^-value above 0.39 (Table 3). However, all the MIFs that were positively associated with lower CH_4_ inhibition in the scatter plot analysis (Figure 6) and significantly varied by the *t*-test and volcano plot between the two sample groups of the study were expected to affect methanogenesis with different levels of contribution. Among these MIFs, the highest Pearson correlation coefficients and relationship values (R^2^-value) of CH_4_ production with MIF intensity were obtained in MIF 15.00_487.2319 (0.75; 0.64) and MIF 15.00_487.2319 (0.69; 0.59), respectively (Table 3; Figure 6). The MIFs selected for their contribution to the lower methane inhibition characteristics of the accessions were included in both sample groups of the study with different levels of intensity. Hence, their variations on the antimethanogenesis effect might be associated with differences in concentration among accessions and within samples of the accessions.

The seven MIFs selected for their contribution to lower CH_4_ inhibition were also analyzed for their correlation and relationship with TGP, *IVOMD*, and CH_4_ per unit of OM digestibility (Table 3). Thus, all seven MIFs were correlated positively and significantly with TCH_4_P, TGP, and CH_4_/*IVOMD* (*p* < 0.05). However, they did not (*p* > 0.05) correlate with *IVOMD* of the substrate feed. Relationship values (R^2^-values) of 0.6929 and 0.0798 were obtained from the scatter plot analysis of MIF 9.06_443.2317 and MIF 15.00_487.2319 intensities with TGP across samples of the study. These MIFs also positively and significantly (*p* < 0.01) correlated with methane yield per OM digested and showed a relationship (R^2^-value) of 0.4812 and 0.5586, respectively. However, the non-significant correlation of these MIFs intensities with *IVOMD* proved that they did not negatively affect fermentation kinetics and productivity of the animals. Therefore, the MIFs selected for their contribution to lower inhibitors of the accessions may have an antagonistic effect on the MIFs that are involved in higher methane inhibition or may stimulate methanogens during ruminal fermentation with no effect on OM digestibility. However, this study did not investigate enough evidence of the bioactivity and mechanism of action of the selected MIFs. The seven *m*/*z* ion-features would have generally been selected as potential candidates, individually or combined, and might be the responsible MIFs, although MIF 9.06_443.2317 and 15.00_487.2319 could be the most promising ion-features for the lower methane inhibitor Moringa accessions. However, identification of their names, structure, and detailed biological activities will be needed in the future.

## 4. Discussion

Plant secondary metabolites are produced by stress and defense response signaling in plant growth [21], though their composition and concentration are determined by the species, physiology, developmental stage, and environmental factors such as light, temperature, water, soil fertility, and salinity [58,59,60]. However, some metabolite ion-features in the current study were not found in all samples and others varied in their intensity, which confirmed the variations of the accessions in composition and concentration, even when they were grown in the same environment. Among the 10 potential candidate metabolite *m*/*z* ion-features selected after screening (three contributed to higher CH_4_ inhibition and seven to lower CH_4_ inhibition), all were included in both sample groups of the study with different concentration levels. However, the intensity of some MIFs within the group was below the limits of detection and recorded a zero value in the detector. Similarly, among the 122 metabolite constituents identified in a previous study of Moringa accessions grown in China and India, 118 were shared components. However, four compounds were detected in only one of the accessions [32]. 

The bioactive metabolites denoted by *m*/*z* ion-features in this study affected methanogenesis in different ranges by suppressing the bioactivity of active compounds involved in CH_4_ inhibition or by changing the pathways that encourage methane production. Thus, the MIFs in higher methane inhibitors reduced methane per unit of organic matter (methane yield) because of their effect on increasing OM digestibility or not having an effect on OM digestibility. On the other hand, an increased total methane production and methane yield were found in the lower methane inhibitor accessions compared with the higher inhibitor group. However, all samples reduced the total CH_4_ production and CH_4_ yield per unit of organic matter digestibility with improved OM digestibility of the substrate feed compared with the control, and the magnitude of antimethanogenic effect varied within accessions. This might be because of the cumulative effect of all the compounds in the sample. Thus, the biological activity of the *m*/*z* ion-features that contributed to higher inhibition might have dominated those that were responsible for lower CH_4_ inhibition. The benefit of decreasing total methane production and methane yield per OM digestibility were recorded in all accessions with a significant improvement in OM digestibility. Thus, it is apparent that this antimethanogenic variation and improvement in OM digestibility are attributed to the difference in the compounds that existed in the plants and their extracts. This is supported by several studies in which the SPM were reported as antioxidant and antimicrobial agents that act against bacteria, protozoa, and fungi [3,20,33,61,62,63,64]. 

The effects of *m/z* ion-features in samples of the study might be attributed to the variation in the dose-dependent influence of the metabolites and their thresholds of maximum and minimum activities [65,66,67]. There were variations within the metabolites in their minimum inhibitory concentration to rumen microbes, and among microbial species in their sensitivity to metabolite action. This might be part of the reason that the MIF 4.53_433.1112 level of intensity was very low, whereas it exhibited comparable and higher antimethanogenesis with the *m*/*z* ion-features having larger intensity. Of the 10 potential *m*/*z* ion-features selected (three for higher methane inhibition and seven for lower inhibition), all were found in both high and low inhibition sample groups. Thus, their variation on the effect of antimethanogenesis might be associated with the level of concentration in the samples. Consequently, along with the type, structure, and functional group of the metabolites affecting their antimethanogenic potential, the concentration of the specific *m*/*z* ion-feature also influenced their effect on CH_4_ production substantially.

The variations in CH_4_ inhibition may also be associated with differences in selective direct inhibition and toxic effects (for example, condensed tannin on methanogens or saponins on protozoa) of the compounds, or depression of the microbial metabolic processes involved in methanogenesis [18,68,69]. Some metabolites such as essential oils and tannins reduce methane production through the depression of rumen fermentation [70,71,72]. However, this type of nonspecific action of the SPM is less important, because it results in lower efficiency of feed utilization and ultimately reduces the productivity of animals. However, all the selected MIFs for higher CH_4_ inhibition potential and for lower methane inhibitors did not reduce the *IVOMD* of the feed significantly. Hence, they would not affect the productivity of ruminant animals negatively during their ultimate application. Furthermore, volatile fatty acids can also be used as a substrate during methanogenesis by methanogens [73]. Hence, the mechanism of metabolites actions also varied in the manipulation of diverting hydrogen (H_2_) into propionate production via lactate, fumarate, and malate pathways, which decrease the flow of H_2_ into CH_4_ production; whereas, some metabolites are involved in favoring H_2_ into the acetyl CoA and formate pathway, which produces more acetate and may increase CH_4_ [74]. However, the biological activities of most SPM are generally associated with the potential of the specific metabolite intruding into the bacterial cell membrane and disintegrating the membrane structure that causes ion leakage. This, in turn, formed irreversible complexes with cholesterol in the protozoal cell membrane [3,20], which ultimately reduced CH_4_ production. Therefore, the observed higher CH_4_ inhibition without a negative effect on the OM digestibility indicates that the inhibition potential obtained in these accessions might be partly associated with changing the H_2_ pathway towards propionate production, or may play a role in the direct inhibition of the methanogens involved in methane production. This feature increases the interest of the MIFs selected in this study; however, further detailed investigations are warranted to annotate these *m*/*z* ion-features and elucidate their pathway and/or mechanism of action on methane production. 

## 5. Practical Applications and Future Research Perspectives

To produce healthy and safe animal products free from long-term residual effects of synthetic feed additives, and for efficient ruminant animal production-friendly with the environment, the study results have several practical implications and future research perspectives. The study approach can be used as an initiative to investigate more effective bioactive metabolites from different medicinal plants. The selected bioactive *m*/*z* ion-features in the study will be used as markers for the selection and standardization of commercial Moringa varieties that will be used as a source of plant extracts to mitigate enteric methane from ruminants. However, further detailed investigations are warranted to annotate these *m*/*z* ion-features and elucidate their pathway and/or mechanism of action on methane production before their application to the mitigation strategies. Besides, their effectiveness in live animals and applicability at the farmers’ level needs more future research.

## 6. Conclusions

The study confirmed that there are variations of *M. oleifera* accession plant extracts in *m*/*z* ion-features intensity, composition, and antimethanogenesis (18%–29%) even when grown in the same environment: the subtropical climate of Pretoria, Gauteng Province, South Africa. However, several secondary plant *m*/*z* ion features were detected in the initial stage of screening; three *m*/*z* ion-features for high CH_4_ inhibition and seven for low CH_4_ inhibition, individually or combined, are potentially contributing to the antimethanogenic variation in the studied accessions. Among them, the bioactive *m*/*z* ion-features 4.53_433.1112 and 4.44_609.1462 were for higher CH_4_ inhibition, whereas the *m*/*z* ion-features 9.06_443.2317 and 15.00_487.2319 for lower CH_4_ inhibition were selected as potential markers for Moringa. The promising *m*/*z* ion-features selected for both higher and lower methane inhibitors did not also adversely affect the fermentation and productivity of the animals. Hence, the use of these *m*/*z* ion-features as potential markers to standardize and select through breeding varieties for high methane inhibition characteristics will increase the benefit without compromising the feed quality when used as dietary additives in ruminant feeding. However, further detailed investigations on matching their name, structure, and biological activities need to be confirmed in the future using a multidisciplinary approach before the application in methane mitigation strategies.

## Figures and Tables

**Table 1:** In vitro fermentation characteristics of Eragrostis curvula hay treated with the leaf extracts of selected *Moringa oleifera* accessions.

Accession	CO	Sample Code	TGP	TCH_4_P	% *IVOMD*	% CH_4_ Inhibition	CH_4_ *IVOMD*	CH_4_ IG
Bulk (A1)	Kenya	A01p10_1	173.71	5.37	59.99	15.72	8.95	low
		A01p10_2	170.03	5.13	59.62	19.42	8.61	low
		A01p16_1	171.97	5.35	58.76	15.97	9.11	low
		A01p16_2	170.28	5.44	60.13	14.55	9.05	low
		A01p32_1	168.82	5.02	59.09	21.20	8.49	low
		A01p32_2	168.28	5.06	58.41	20.64	8.65	low
Mean ± SEM			170.51 ± 1.23 ^B^	5.23 ± 0.13 ^B^	59.33 ± 0.38 ^A^	17.92 ± 2.01 ^B^	8.81 ± 0.18 ^B^	
07229 (A2)	Kenya	A02p09_1	175.14	5.39	60.39	15.42	8.92	low
		A02p09_2	174.59	5.42	60.22	14.96	8.99	low
		A02p23_1	171.93	5.31	58.07	16.70	9.14	low
		A02p23_2	173.25	5.28	59.16	17.15	8.92	low
		A02p34_1	170.29	4.93	59.29	22.54	8.32	low
		A02p34_2	170.65	4.97	62.78	22.03	7.91	low
Mean ± SEM			172.64 ± 1.55 ^B^	5.21 ± 0.15 ^B^	59.99 ± 0.88 ^A^	18.13 ± 2.35 ^B^	8.70 ± 0.36 ^B^	
07633 (A3)	Mali	A03P11_1	163.61	4.64	58.98	27.20	7.86	high
		A03P11_2	162.81	4.68	60.16	26.57	7.77	high
		A03P19_1	161.43	4.69	58.64	26.42	7.99	high
		A03P19_2	162.31	4.73	59.22	25.77	7.98	high
		A03P31_1	157.05	4.22	62.05	33.69	6.81	high
		A03P31_2	157.53	4.42	61.18	30.55	7.23	high
Mean ± SEM			160.79 ± 2.20 ^C^	4.56 ± 0.14 ^C^	60.04 ± 0.99 ^A^	28.37 ± 2.20 ^A^	7.61 ± 0.37 ^C^	
Pretoria (A11)	South Africa	A11P01_1	164.31	4.52	56.3	29.10	8.02	high
		A11P01_2	165.19	4.49	55.73	29.56	8.05	high
		A11P15_1	163.57	4.77	58.4	25.17	8.16	high
		A11P15_2	164.19	4.79	59.34	24.75	8.08	high
		A11P33_1	163.65	4.32	58.25	32.26	7.41	high
		A11P33_2	163.71	4.25	57.51	33.22	7.40	high
Mean ± SEM			164.10 ± 0.40 ^BC^	4.52 ± 0.16 ^C^	57.59 ± 1.02 ^B^	29.01 ± 2.48 ^A^	7.85 ± 0.28 ^C^	
Control			203.84 ± 5.32 ^A^	6.37 ± 0.18 ^A^	54.68 ± 0.38 ^C^		11.25 ± 0.40 ^A^	

The mean values of accessions with different superscript letters along the column were significantly (*p* < 0.01) different. CO: country of origin; TGP: total gas production (mL/g DM); TCH4P: total methane production (mL/g DM); *IVOMD*: in vitro organic matter digestibility; A1P10_1 in the sample code referring to accession 1, plot 10, sample 1; A1P10_2: accession 1, plot 10, sample 2, etc.; SEM: standard error of mean; CH4 IG: methane inhibition group; CH4/*IVOMD*: methane yield per unit of organic matter digestibility.

**Table 2:** Summary data of potential *m*/*z* ion-features contributed to higher methane inhibition in Moringa accessions and their Pearson correlation with major in vitro fermentation characteristics.

	*m*/*z* Ion-Features (MIFs)		
	Negative Ion Mode	Positive Ion Mode	
	4.44_609.1462	4.53_433.1112	14.22_682.3577
Detected mass	609.1462	433.1112	682.3577
Actual mass	609.1462	432.1112	681.3577
RT	4.44	4.53	14.22
ALI	63.89	1.81	1.54
AHI	167.66	4.10	2.83
Fold change	2.02	2.54	2.13
*p*-value	0.021	4.7 × 10^−7^	0.0047
Pearson correlation of the selected MIFs with TCH_4_P, TGP and *IVOMD* (2-tailed)			
TCH_4_P	−0.48853 *	−0.73828 **	−0.50434 *
TGP	−0.63255 **	−0.79327 **	−0.36979 *
*IVOMD*	0.08915	−0.29749	−0.45888 *
CH_4_/*IVOMD*	−0.58925 **	−0.65649 **	−0.36577

** Significantly correlated at *p* < 0.01; * Significantly correlated at *p* < 0.05. FC: fold changes; actual mass: detected mass minus one hydrogen atom (in the positive ion mode); RT: retention time; ALI: average intensity of the *m*/*z* ion-features in the lower methane inhibition sample group; AHI: average intensity of the *m*/*z* ion-features in the higher methane inhibition sample group; TCH_4_P: total methane production (ml/g DM); TGP: total gas production (mL/g DM); *IVOMD*: in vitro organic matter digestibility (%).

**Table 3:** Summary data of potential candidate *m*/*z* ion-features contributed to lower methane inhibition in the accessions and Pearson correlation with in vitro fermentation characteristics.

	*m*/*z* Ion-Features						
	Negative Ion Mode	Positive Ion Mode					
	11.01_265.1448	9.06_443.2317	11.84_581.3658	12.31_355.1513	14.47_341.2650	15.00_487.2319	15.02_385.2907
Detected mass	265.1448	443.2317	581.3558	355.1513	341.2650	487.2319	385.2907
Actual mass	265.1448	442.2317	580.3658	354.1513	340.2650	486.2319	384.2907
RT	11.01	9.06	11.84	12.31	14.47	15.00	15.02
ALI	668.19	157.22	3.53	18.91	57.35	214.01	84.97
AHI	394.07	58.52	1.81	5.15	15.87	33.12	19.11
Fold change	0.32	0.39	0.48	0.26	0.26	0.14	0.21
*p*-value	3.2 × 10^−6^	4.75 × 10^−7^	2.9 × 10^−4^	1.1 × 10^−4^	4.2 × 10^−4^	4.27 × 10^−6^	1.2 × 10^−4^
Pearson correlation of the selected MIFs with TCH_4_P, TGP and *IVOMD* (2-tailed)							
TCH_4_P	0.615 **	0.768 **	0.653 **	0.693 **	0.632 **	0.802 **	0.741 **
TGP	0.662 **	0.648 **	0.509 *	0.580 **	0.520 **	0.675 **	0.637 **
*IVOMD*	0.154	0.264	0.439	0.294	0.325	0.177	0.317
CH_4_/*IVOMD*	0.578 **	0.694 **	0.523 **	0.611 **	0.531 **	0.747 **	0.655 **

The MIF intensities between higher and lower methane inhibition sample groups were significantly different at the corrected *p* ≤ 6.25 × 10^−3^ in the negative and *p* ≤ 6.4 × 10^−4^ in the positive ion mode. Pearson correlation: ** significant at *p* < 0.01 or * significant at *p* < 0.05 (2-tailed). RT: retention time; ALI: average intensity of the *m*/*z* ion-features in the lower methane inhibition sample group; AIH: average intensity of the *m*/*z* ion-features in the higher methane inhibition sample group; actual mass: detected mass minus one hydrogen atom (in the positive ion mode); TGP: total gas production (mL/g DM); TCH_4_P: total methane production (mL/g DM); *IVOMD*: in vitro organic matter digestibility (%).

## Data Availability

Data will be stored in the University of Pretoria repository, and access to the data will be granted by making a reasonable request to the University of Pretoria or the corresponding author.
